# PANoptosis-related molecular clustering and prognostic signature associated with the immune landscape and therapy response in breast cancer

**DOI:** 10.1097/MD.0000000000039511

**Published:** 2024-09-13

**Authors:** Yiming Cao, LinJing Guan, Li Yang, Changyuan Wei

**Affiliations:** a Department of Breast Surgery, Guangxi Medical University Cancer Hospital, Nanning, P.R. China; b Department of Breast and Thyroid Surgery, Liuzhou People’s Hospital, Liuzhou, P.R. China; c Department of Abdomen Ultrasound, Nanning Sixth People’s Hospital, Nanning, P.R. China; d Department of Pathology, Liuzhou People’s Hospital, Liuzhou, P.R. China.

**Keywords:** breast cancer, drug sensitivity, PANoptosis, prognostic signature, tumor microenvironment

## Abstract

Breast cancer (BC) remains one of the most pervasive and complex malignancies. PANoptosis represents a recently identified cellular mechanism leading to programmed cell death. However, the prognostic implications and influence on the immune microenvironment of BC pertaining to PANoptosis-related genes (PRGs) remain significantly understudied. We conducted differential expression analysis to identify prognostic-Related PRGs by the Cancer Genome Atlas (TCGA) and the Gene Expression Omnibus (GEO) databases. Next, we identified the PANoptosis-related molecular subtype using the consensus clustering analysis, and constructed and validated the PANoptosis-related prognostic signature using LASSO and Cox regression analyses. ROC curves were employed to assess the performance of the signatures. Furthermore, drug sensitivity between low- and high-risk group were analysis. Finally, we conducted RT-qPCR to assess the gene expression levels involved in this signature. We categorized BC patients into 2 distinct molecular clusters based on PRGs and identified differentially expressed genes associated with prognosis. Subsequently, BC patients were then divided into 2 gene clusters. The identified PRGs molecular clusters and gene clusters demonstrated association with patient survival, immune system functions, and biological processes and pathways of BC. A prognostic signature comprising 5 genes was established, and BC patients were classified into low- and high-risk groups based on the risk scores. The ROC curves demonstrated that those in the low-risk category exhibited notably extended survival compared to the high-risk group. A nomogram model for patient survival was constructed based on the risk score in conjunction with other clinical features. High-risk group had higher tumor burden mutation, CSC index and lower StomalScore, ImmuneScore, and ESTIMATEScore. Subsequently, we established a correlation between the risk score and drug sensitivity among BC patients. Finally, qRT-PCR results showed that the expression of CXCL1, PIGR, and TNFRSF14 significantly decreased, while CXCL13 and NKAIN were significantly increased in BC tissues. We have developed a molecular clustering and prognostic signature based on PANoptosis to improve the prediction of BC prognosis. This discovery has the potential to not only assist in assessing overall patient prognosis but also to deepen our understanding of the underlying mechanisms of PANoptosis in BC pathogenesis.

## 1. Introduction

Breast cancer (BC) remains one of the most pervasive and complex malignancies affecting millions globally. In 2020, 2 million new cases of BC were diagnosis in women globally.^[1]^ In addition, the incidence of BC is increasing by 0.5% every year.^[2]^ BC is a multifaceted disease characterized by a myriad of molecular and pathological diversities, leading to varied prognosis and therapeutic responses.^[3]^ Previous study suggested that breast cancer were divided into 4 molecular subtypes based on estrogen receptor (ER), progesterone receptor (PR), human epidermal growth factor receptor 2 (HER2) and cell proliferation marker Ki-67. These subtypes including Luminal A, Luminal B, HER2-enriched, and triple-negative.^[4]^ The treatment of breast cancer depends on different molecular subclasses, and its treatment pathways include local/regional interventions such as surgery and radiation therapy, as well as systemic therapy. Systemic therapy includes hormone therapy for hormone-positive cases, chemotherapy, anti-human epidermal growth factor receptor 2 (HER2) therapy for HER2-positive cases, and the integration of immunotherapy.^[4,5]^ But the combined results of these treatments are still unsatisfactory. Studies have shown a strong correlation between programmed death and the onset and prognosis of BC.^[6]^ Cell death often occurs in the central region of solid tumors of BC, so programmed death may become a new approach to the integrated treatment of solid BC, including various subtypes.^[7]^In recent years, increased attention has been devoted to understanding the intricacies of cell death mechanisms in cancer, as they are instrumental in the progression, treatment, and recurrence of the disease. As time goes on, recent research has shown that higher parity, the use of tamoxifen, and having a first child at an early age can decrease the overall lifetime risk of developing BC.^[8–10]^ Simultaneously, nipple-sparing mastectomy, lymphatic mapping, MRI, neoadjuvant systemic therapy, partial breast irradiation, and adjuvant therapy can enhance the outlook for BC patients.^[11]^ The main cause of tumor invasiveness is BC stem cells, and the study of breast stem cell and genes is also the main challenge of cancer treatment.^[12]^ Presently, the clinical, pathological, and molecular characteristics in our knowledge base fall short in accurately forecasting patient prognosis. Consequently, there remains an urgent demand for novel prognostic markers to assess patient outcomes and steer treatment strategies.

PANoptosis, a novel, intricate cell death mechanism that integrates elements of apoptosis, necroptosis, and pyroptosis, was recently proposed. Distinct morphological features and energy-dependent biochemical mechanisms often have distinct processes of programmed cell death or apoptosis, including apoptosis, pyroptosis, and necrosis, which is necessary for normal cell turnover and tissue homeostasis.^[13]^ Apoptosis is characterized by a series of distinctive morphological alterations in cell structure and enzyme-driven biochemical processes. This unique feature allows for the removal of cells from the body with minimal damage.^[14]^ Earlier studies on cell death highlighted the unique regulation of each of apoptosis, pyroptosis, necroptosis, and ferroptosis pathways, but recent studies have emphasized on the redundancies and crosstalk among them. PANoptosis, which highlights the crosstalk and coordination between pyroptosis, apoptosis, and necroptosis is a recently proposed concept. Malireddi RKS et al found that the innate immune sensor ZBP1 and the essential cell survival kinase TAK1 play an important role in the regulation of RIPK1/RIPK3–FADD–caspase-8 cell death complex assembly. Its versatility in executing Pyroptosis, Apoptosis, and Necroptosis, which we dubbed here as PANoptosis.^[15]^ Some research shown that PIPK1, ZBP1, PIPK3 and CASP8 would be gotten through the regulation of PANoptosis to homeostasis, cell death, and inflammatory immune responses.^[16]^ Currently, PANoptosis has been investigated in various cancer diseases, and the findings have shown the potential for predicting PANoptosis and the intratumorally immune landscape in patients through PANoptosis-based molecular clustering and prognostic signatures.^[17,18]^ As of now, there is a lack of research on BC that utilizes PANoptosis-based molecular clustering and prognostic signatures.

Our current study is centered on investigating the role, significance, therapeutic possibilities, and prognostic value of PANoptosis-associated genes (PRGs) and pathways in molding the tumor microenvironment, impacting immune responses, and influencing patient outcomes in the context of BC. Adopting a comprehensive approach that integrates robust data collection, advanced analytical methods, and multifaceted assessment of the tumor landscape to uncover the relationship between PANoptosis and BC, which may help our understanding BC PANoptosis mechanism and design new treatment for BC. In addition, we also used qRT-PCR to validated the signature genes in BC.

## 2. Materials and methods

### 2.1. Data collection

The BC patient data, encompassing clinical records, RNA sequencing data, and mutation profiles, were sourced from publicly available databases, including The Cancer Genome Atlas (TCGA) at https://portal.gdc.cancer.gov/ and the Gene Expression Omnibus (GEO). The datasets were rigorously curated to include comprehensive clinical annotations and follow-up information. Patients without follow-up data or those with incomplete clinical information were excluded from this study. 1118 BC patients and 113 normal samples of RNA-sequencing data were enrolled from TCGA, and 61 BC RNA-sequencing data were download from GSE37751, were used in this analysis. BC of TCGA and GEO transform fragments per kilobase million (FPKM) into transcripts per million (TPM) by R software.^[15,19,20]^ Tumor samples from both TCGA and GEO datasets were combined, and the “sva” R package was used to correct for batch effects. 29 PRGswere collected from previous studies,^[21,22]^ and the details are presented in Table S1, http://links.lww.com/MD/N484. Clinical information for BC patients is provided in Supplemental Table S2, http://links.lww.com/MD/N484.

### 2.2. Differential expression analysis and identification of prognostic-related PRGs

To identify differential expression levels of PRGs between BC patient and non-tumor patient, we performed a Wilcoxon rank-sum test for differential analysis. Subsequently, we employed Kaplan–Meier (KM) analysis and univariate Cox regression to further assess the PRGs associated with prognosis.

### 2.3. Analysis of somatic mutation and copy number variation

Using the “limma” package, we extracted the expression levels of PRGs in both normal and BC samples. The threshold for differential analysis was set at |fold change| > 1, with *P* < .05. Copy number variation (CNV) data and somatic mutation files in maf format for HCC samples were retrieved from the TCGA database. We employed the R package “circos” to visualize the genomic locations of RRGs based on the gene reference file. To identify tumor mutations, we generated waterfall plots of PRGs and 2 risk score groups in BC using the “maftools” R package.

### 2.4. PRG consensus clustering analysis

PRGs consensus cluster analysis was conducted to classify BC patients into different PANoptosis related subgroups of BC. We used the “ConsensusClusterPlus” package in R to perform unsupervised clustering based on the expression profiles of PRGs of PC patients. The Euclidean distance was chosen as the method to calculate the distance. The seed was set to 123,456 for PAM algorithm clustering analysis. Kaplan–Meier (KM) method and log-rank test were used to compare the prognostic factors between the 2 groups. The optimal number of clusters was asses using the consensus matrix and the cumulative distribution function (CDF) curve. Principal component analysis (PCA) was performed to validated the existence of distinct subgroups. Differential expression genes (DEGs) and differences in clinical characteristics were analyzed by Wilcoxon test using “limma” package. In multiples | log fold change (FC) | >0.585; DEGs were screened as the threshold value. *P* value < 0.05. Gene set variation analysis (GSVA) was used to analyze the differences in biological processes conducted by “gsva” R package.

We also used the single sample gene set enrichment analysis (ssGSEA) to calculate the immune cell infiltration score and evaluate the immune activity.

### 2.5. Functional enrichment analysis of DEGs

Functional enrichment analysis, including gene ontology (GO) and Kyoto Encyclopedia of Genes and Genomes (KEGG) pathway analyses, were conducted to elucidate the biological pathways and functions associated with these DEGs. The threshold was set at *P*-value < 0.05.

### 2.6. Establishment of prognostic features based on PANoptosis

To identify PANoptosis-Related Differentially Expressed Genes (PRDEGs), we utilized univariate Cox regression analysis, enabling the selection of additional PANoptosis-related genes for constructing a prognostic signature. BC patients were stratified into 2 distinct geneclusters based on the expression of PRDEGs. The PRGs expression, clinicopathological characteristics and survival time of the 2 geneclusters of BC patients were compared. Additionally, the least absolute shrinkage and selection operator (LASSO) regression analysis was utilized to mitigate the risk of overfitting among PRDEGs.^[17]^ Finally, we conducted multivariate Cox regression analysis to identify the top 5 risk genes and establish the PANoptosis-related prognostic model. PANoptosis score, where n represent the number of risk genes, exp (Xi) represents the expression level and coef (Xi) represents the coefficient. Based on the median risk score, BC patient was stratified into high- and low-risk groups ALL, training set, and test set. The disparity in survival time between high-risk and low-risk patients was assessed using Kaplan–Meier (KM) analysis, and the receiver operating characteristic (ROC) curve and area under the curve (AUC) was utilized to evaluate the signature’s accuracy. Moreover, a nomogram model was established integrating risk scores derived from the prognostic signature along with pertinent clinical factors. Calibration graphs were generated to visually represent the distinctions between the predicted and actual survival rates among BC patients.

### 2.7. Assessment of the Tumor Microenvironment

In our study, CIBERSORT was utilized to estimate the proportions of immune cells infiltrating both high-risk and low-risk subgroups. Our analysis provided insights into the relevance of these genes to the risk model and the tumor microenvironment (TME). We also investigated the relationship between 2 prognostic genes and the quantity of infiltrated immunocytes. Additionally, we analyzed the correlation between the abundance of immune cells and the 5 genes in question. Finally, we compared the TME scores, including stromal, immune, and ESTIMATE scores, between the high-risk and low-risk groups.

### 2.8. Relationships of cancer stem cell, tumor mutational burden (TMB), and somatic mutations in distinct PRGs risk score groups

The mutation annotation format (MAF) analyssed through the “maftools” R package, and the mutation data were analyzed to identify significantly mutated genes in the high-risk and low-risk subgroups. Correlation between risk scores and tumor mutation burden (TMB) scores and cancer stem cell (CSC) index were discussed by Spearman’s method.

### 2.9. Effects on immune therapy and chemotherapeutics

To calculate the drug responses of BC patients in high- and low-risk group, we evaluated 198 drugs sourced from the Genomics of Drug Sensitivity in Cancer v2 (GDSC) database, which can be accessed at https://www.cancerrxgene.org/, using the “OncoPredict” R package.^[23]^

### 2.10. Real-time quantitative PCR (RT-qPCR)

We collected 21 pairs of BC tissues and corresponding non-tumor tissues from patients at LiuZhou people’s hospital affiliated to Guangxi medical university. In the present study, the enrolled BC samples were not classified as the subtypes. These samples were diligently preserved at -80°C. The study was conducted in accordance with the approval of the Ethics Committee of LiuZhou people’s hospital affiliated to Guangxi medical university, and all participants provided informed consent.

We extracted total RNA from these tissues using Trizol. Next, extracted RNA was then transcribed into cDNA (Thermo Fisher Scientific, M16325) and used cDNA for real-time quantitative PCR (Thermo Fisher Scientific, A25742). To determine relative expression levels, we employed the 2−ΔΔCt method with β-actin as the reference gene (Supplemental Figure S1, http://links.lww.com/MD/N484). The primer sequences used for PCR amplification are presented in Supplemental Table S3, http://links.lww.com/MD/N484. We then assessed the differences in expression levels between BC tissues and adjacent non-tumor tissues using unpaired t-tests.

### 2.11. Ethical review

The research received approval from the Ethics Committee of LiuZhou people’s hospital affiliated to Guangxi medical university, and informed consent was obtained from all participants.

### 2.12. Statistical analysis

Data analysis and visualization were conducted using R (Version 4.2.2) and GraphPad Prism 9 software. We initially compared differences in data between 2 groups that followed a normal distribution using the t-test. For data that did not conform to a normal distribution, we used the Wilcoxon rank-sum test for comparisons. To examine correlations, we employed Spearman or Pearson analysis as appropriate. A significance level of *P* < .05 was considered statistically significant.

## 3. Result

### 3.1. Differential expression and genetic alterations of PRGs in BC

**Figure 1.**
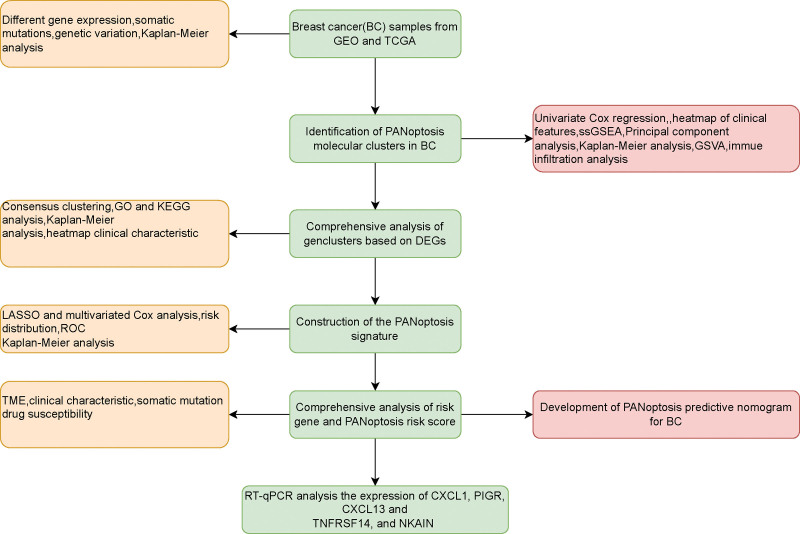
The flowchart of this study.

**Figure 2.**
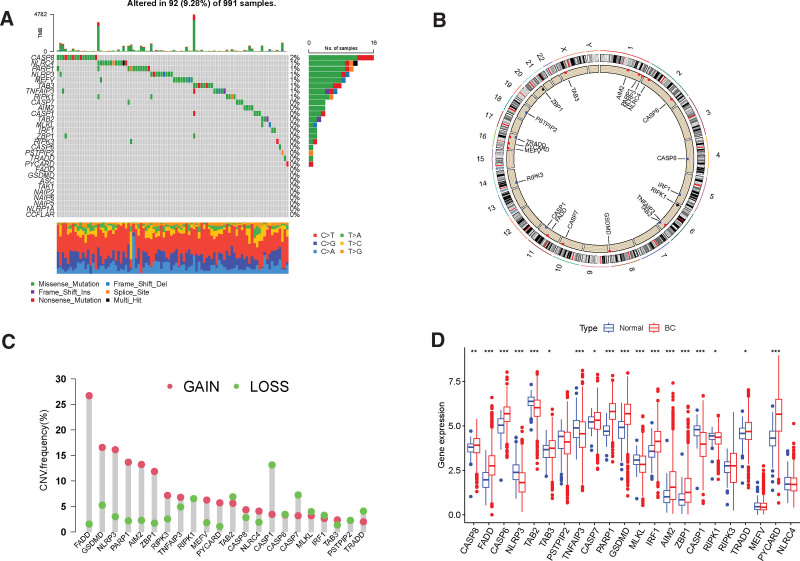
Expression and genetic alteration of PRGs in breast cancer: (A) Mutation situations of 29 PRGs. (B) Locations of CNV alterations for PRGs on 23 chromosomes. (C) Copy number alterations of PRGs. (D) Expression difference of PRGs. *P < .05; **P < .01; ***P < .001. CNV = copy number variation, PRGs = PANoptosis-related genes.

The flowchart of this study is shown in Figure 1. 1118 BC patient and 113 normal sample expression data were downloaded from the TCGA-BRCA database, and the PRGs expression between these 2 groups were compared. 29 PRGs from previous published studies were enrolled in this study for analysis. Figure 2A shown that 92(9.28%) of 991 sample had PRGs somatic mutations. CASP8 demonstrated the highest mutation frequency among the PRGs. Figure 2B illustrate the chromosomal locations of CNV alterations in PRGs. We then conducted an analysis of CNV for the PRGs, and identified FADD GSDMD, NLRP3, PARP1 AIM2, ZBP1, RIPK3, TNFAIP3, MEFV, PYCARD, CASP8, NLRC4, and TAB3 as having higher CNV. Conversely, TAB2, CASP1, CASP7, MLKL, IRF1, PSTPIP2 and TRADD displayed significant CNV decreases (Fig. 2C). We then performed a differential expression gene of PANoptosis analysis between BC and normal sample. The results showed CASP8, FADD, CASP6, TAB3, CASP7, PARO1, GSDMD, IRF1, AIM2, ZBP1, RIPK3, TRADD and PYCARD were upregulated in BC samples, whereas NLRP3, TAB2, PSTPIP2, TNFAIP3, MLKL and CASP1 were downregulated in BC compared with normal sample (*P* < .05) (Fig. 2D).

**Figure 3.**
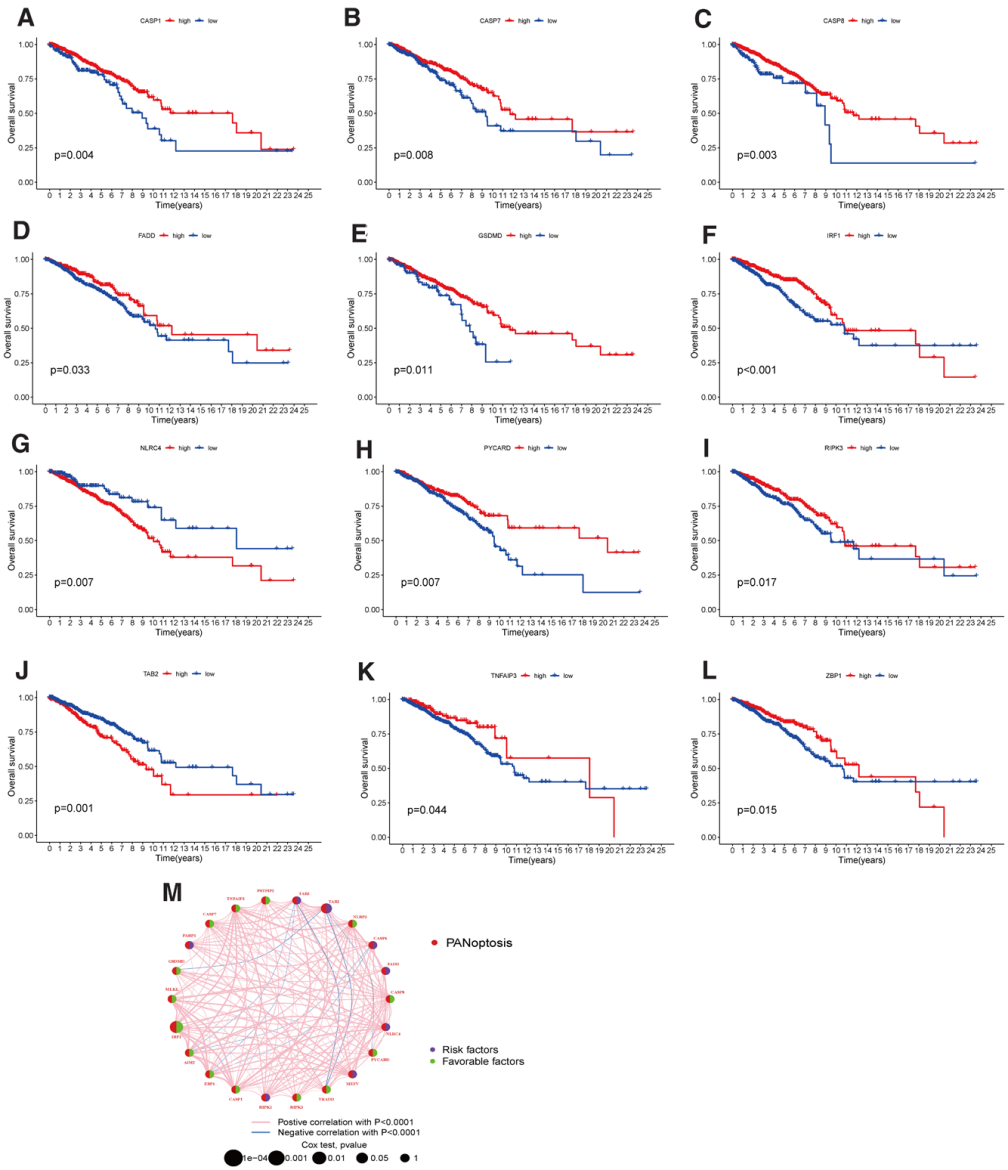
Prognosis significance of PRGs of BC patients in TCGA and GEO in BC: (A–L) The survival curve of CASP1, CASP7, CASP8, FADD, GSDMD, IRF1, NLRC4, RYCARD, RIRK3, TAB2, TNFAIP3, and ZBP1 displays the OS of BC patients. (M) Prognostic network of PRGs. BC = breast cancer, OS = overall survival, PRGs = PANoptosis-related genes.

Subsequently, we conducted KM and Cox analysis to evaluate the prognostic significance of PRGs in BC patients using data from the TCGA and GSE37751 datasets. Our results indicated that 12 was significant associated with overall survival (OS) (Figs. 3A-L). To explore the association and relationship of PRGs and their prognostic significance, a network map was developed (Fig. 3M).

### 3.2. Identification of PANoptosis molecular subtypes

**Figure 4.**
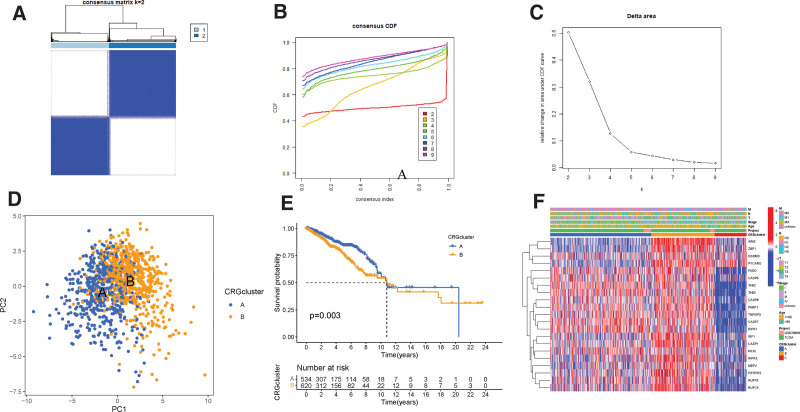
PRG molecular subtypes and their clinicopathological features: (A–C) Identification of 2 molecular subtypes (k = 2) and their correlation area through consensus clustering analysis. (D) PCA showed good distinction between 2 PRGclusters. (E) KM curve indicated that PRGcluster A had longer survival time than PRGcluster B (*P* = .003). (F) Heatmaps showed the relationship between PRGclusters and clinical features and PRGs expression in breast cancer patients. PRGs = PANoptosis-related genes.

Based on PRGs expression in BC, we employed a consensus clustering algorithm to categorize BC patients into 2 distinct groups. Our results indicated that choosing k = 2 was an optimal approach for stratifying patients into 2 groups, which we labeled as molecular subtypes A (n = 535) and B (n = 621) (Figs. 4A-C). Subsequently, Principal Component Analysis (PCA) verified the significant differences in PANoptosis-related transcription profiles between PRGcluster A and B (Fig. 4D). Survival analysis showed that PRGcluster A had a significantly longer OS time than PRGcluster B (ρ = 0.003) (Fig. 4E). Additionally, we profiled the associations between PRGs’ expression and various clinical features, including age, grade, and N stage, with respect to different molecular subtypes of BC (Fig. 4F). The heatmap showed that most PRGs was upregulated in PRGcluster A, featuring genes such as IRF1, CASP1, and ZBP1.

### 3.3. Clinical characteristics of TME in PRGcluster A and B

**Figure 5.**
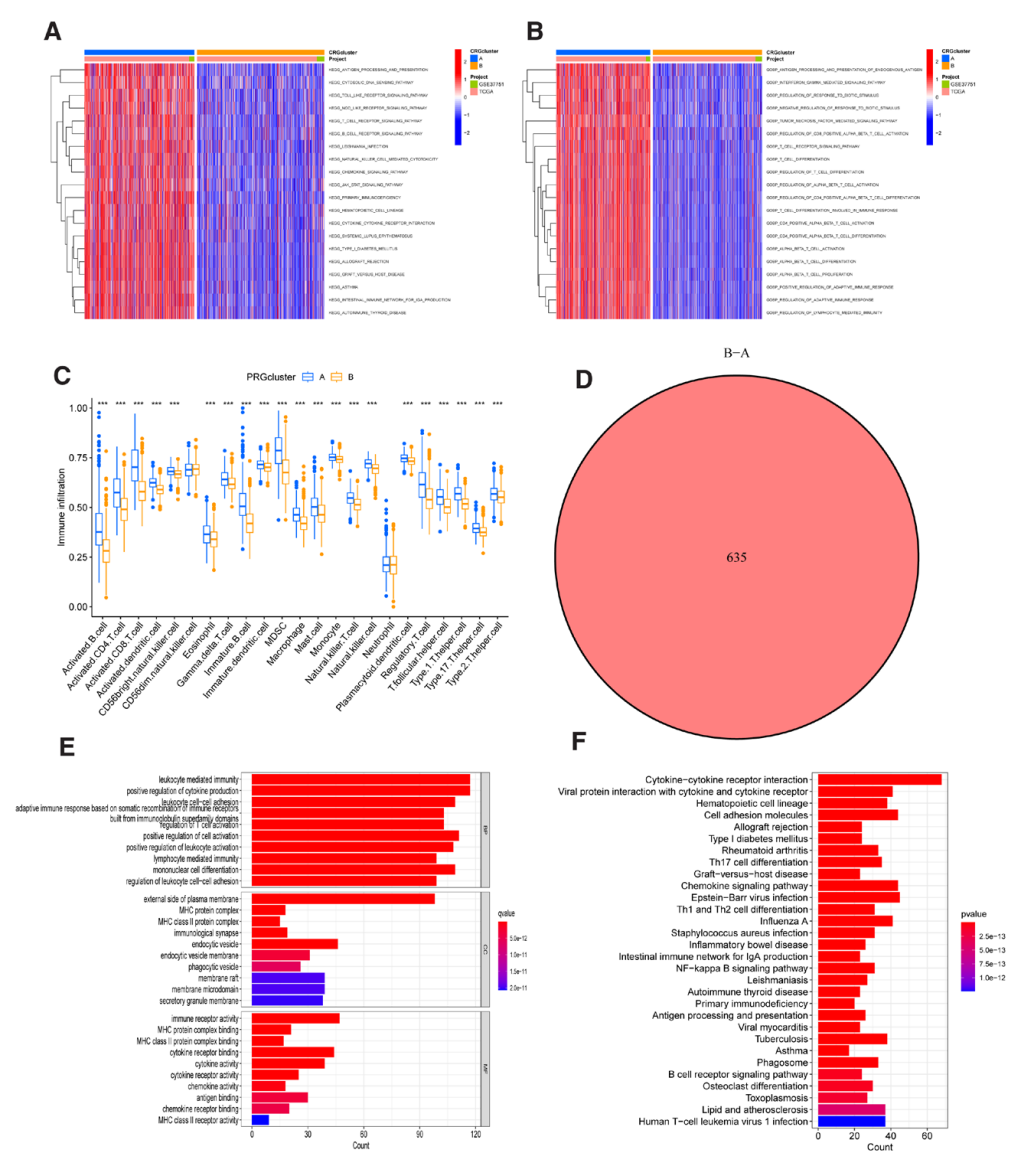
Identification of geneclusters based on differentially expressed genes (DEGs): (A and B) GSVA showed the enriched KEGG pathways in PRGclusters. (C) ssGSEA investigated the differences of immune cell infiltration between 2 geneclusters. (D) The Venn diagram shows the intersection of the differentially expressed PRGs. (E and F) GO and KEGG analyses showed the relevant biological processes (BP), cellular components (CC), molecular functions (MF) and pathways. GO = gene ontology, GSVA = gene set variation analysis, KEGG = Kyoto Encyclopedia of Genes and Genomes, PRGs = PANoptosis-related genes.

To gain deeper insights into the characteristics of the TME within distinct PRGcluster, we conducted a comprehensive analysis of encompassing KEGG and GO GSEA and GSVA enrichment analysis. The KEGG GSVA enrichment highlighted that PRGcluster A exhibited significant enrichment in natural killer cell-mediated cytotoxicity pathway, antigen processing and presentation pathway, primary immunodeficiency, B and T cell receptor signaling pathway, JAK-STAT signaling pathway (Figs. 5A-B).

We then performed ssGSEA analysis to assesses the differences between PRGcluster A and B. ssGSEA results showed that PRGcluster A exhibited higher levels of immune cell infiltration, encompassing activated B cells, MDSC, activated CD4 + and CD8 + T-cells, mast cell, macrophages, activated dendritic cells, and natural killer cells (Fig. 5C).

We then conducted a differential analysis of the 2 PRGclusters to identify differentially expressed genes. The Venn diagram identified 635 DEGs intersection across these 2 clusters (Fig. 5D). GO and KEGG enrichment analysis was carry out to elucidate the potential function and pathway of these 635 DEGs. The GO analysis indicated that the DEGs exhibited close associations with cellular components, including the leukocyte mediated immunity, positive regulation of cytokine production, lymphocyte mediated immunity. In terms of biological processes, these genes were primarily involved in responding to immunological synapse, external side of plasma membrane, endocytic vesicle. Molecular functions exhibited closely related to immune receptor activity, cytokine receptor binding, cytokine activity (Fig. 5E). Additionally, the results of the KEGG enrichment analysis highlighted the involvement of these DEGs in various pathways, including Cytokine-cytokine receptor interaction, Cell adhesion molecules, NF-kappa B signaling pathway, Primary immunodeficiency (Fig. 5F).

### 3.4. Construction of geneclusters based and prognostic risk model

**Figure 6.**
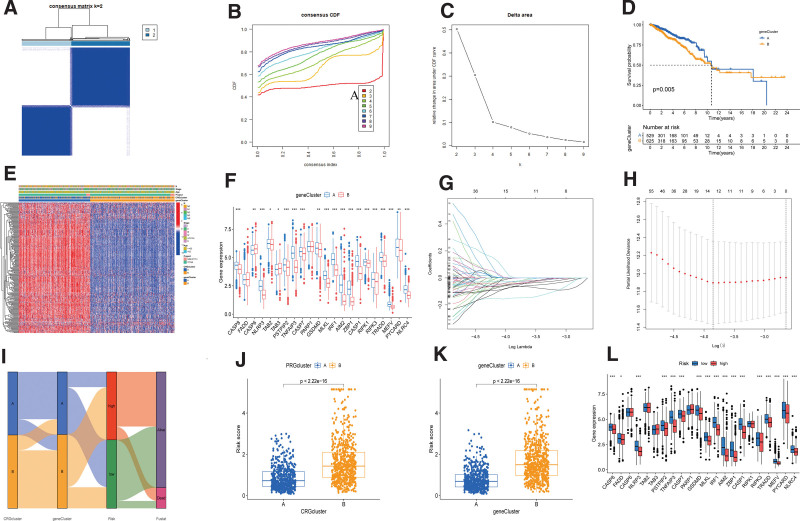
PRG gene subtypes and their clinicopathological features and prognostic risk model: (A–C): Identification of 2 gene subtypes (k = 2) and their correlation area through consensus clustering analysis. (D) KM curve indicated that genecluster A had longer survival time than genecluster B (*P* = .005). (E) Heatmap showed the association between genecluster and clinical features. (F) Expression levels of PRGs in 2 geneclusters. (G and H) The LASSO regression analysis and partial likelihood deviance on the prognostic genes. (I) Sankey plot showed the correlation between molecular classifications, risk groups and survival status in breast cancer patients. (J and K) Association between risk score and molecular and gene classifications. (L) Expression levels of PRGs in 2 risk groups. *P < .05; **P < .01; ***P < .001. PRGs = PANoptosis-related genes.

To further assessed the important role of these DEGs, a univariate Cox regression analysis was conducted. BC patients were divided into genecluster A and genecluster based on the expression levels of these DEGs (Figs. 6A-C). Survival analysis showed that genecluster A showed better OS compared to genecluster B (Fig. 6D). We then constructed a complex cluster-based heatmap by integrating the age, clinical stage information of BC patients (Fig. 6E). The boxplot showed that only CASP6 and TAB3 were downregulated in geneCluster A, whereases most PRGs were upregulated in genecluster A, including CASP8, NLRP3, TAB2, MLKL (Fig. 6F).

We developed a PRG Risk model based on the expression profiles of prognostic DEGs obtained from 2 molecular subtypes. BC patients were randomly divided into training (n = 578) and testing (n = 577) groups at a 1:1 ratio, employing the “caret” package in R. We then conducted a LASSO regression and multivariate Cox analyses to the 635 prognostic DEGs, aiming to mitigate the risk of gene overfitting during signature generation and derive an optimum prognostic signature (Fig. 6G). The multivariate Cox regression analysis ultimately selected best 5 genes for inclusion in the prognostic risk model in training sets: Risk score = (-0.129* expression of CXCL13) + (-0.347* expression of TNFRSF14) + (-0.127* expression of PIGR) + (-0.271* expression of CXCL1) + (-0.126* expression of NKAIN1). Both training set and testing set were divided into low and high-risk groups based on the median score calculated using the risk score formula in training group. Subsequently, all set files were combined by the training set and the testing set files (Fig. 6H). Sankey diagram showed BC patients were divided into 2 PRGclusters, 2 geneclusters, and 2 PRG risk score group (Fig. 6I). The results of boxplot showed that PRGcluster B and genecluster B were significantly associated with higher risk score in BC patients (Figs. 6J-K). The differential analysis of PRGs expression between high- and low-risk group were shown in boxplot of Figure 6L.

### 3.5. Validation of the prognostic risk model

**Figure 7.**
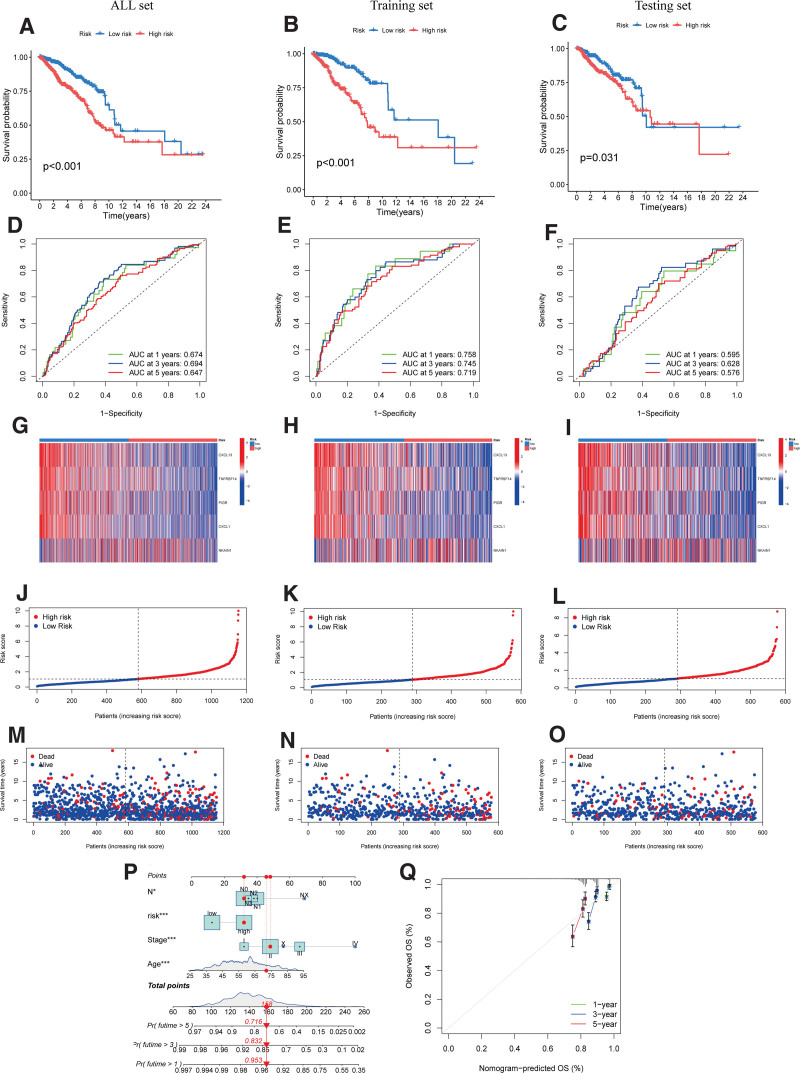
Validation of the prognostic value of the signatures: (A–C) The KM curve of all sets, training set, and testing set, respectively. (D–F) ROCs for 1-, 3-, and 5-year OS prediction of all sets, training set, and testing set, respectively. (G–O) The risk curve consists of genes expression heat map, risk score curves, and survival status point plot of all sets, training set, and testing set, respectively. (P) The nomogram of the risk score and clinical parameters (age, gender, and stage) of all sets. (Q) The calibration curves displayed the accuracy of the nomogram in the 1st, 3rd, and 5th years. OS = overall survival.

The Kaplan–Meier analysis results showed that patients in high-risk group exhibited a significantly lower probability of survival compared to those in the low-risk group in both all, training, and testing set (Figs. 7A-C). To assess the predictive efficacy of the risk score, we generated ROC curves analysis, with AUCs of 0.674, 0.694, and 0.647 for 1-, 3-, and 5-year survival in all set, respectively, indicating the sensitivity and specificity of the risk model (Fig. 7D). For training sets, the AUC for 1-, 3-, and 5-year survival was 0.758, 0.745, and 0.719, respectively (Fig. 7E). For testing sets, the AUC for 1-, 3-, and 5-year survival was 0.595, 0.628, and 0.576, respectively (Fig. 7F). Additionally, we examined the expression differences of the 5 hub genes comprising the signature in the all set (Fig. 7G), training (Fig. 7H), and testing sets (Fig. 7I). The scattergram of PRG Risk score analysis showed that BC patients’ OS time decreased while PRGs score increased (Figs. 7J-O). Based on the risk score and other clinical characteristics, we development a nomograms model to assess the proportional hazards hypothesis in the multivariate Cox model (Fig. 7P). calibration plots were employed to visualize the discrepancies between the predicted and actual survival probabilities of colon cancer patients, indicating the nomogram could predicted the prognosis of BC (Fig. 7Q).

### 3.6. Relationships between the TME and high- and low-risk groups

**Figure 8.**
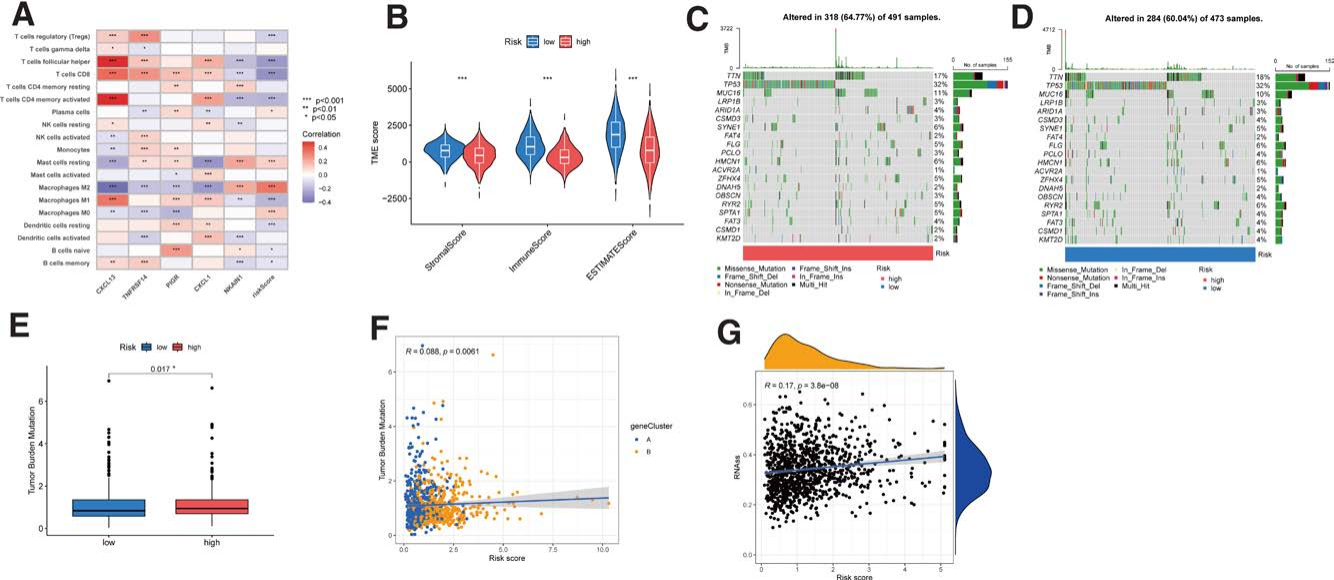
To assess the tumor microenvironment, immune checkpoint genes, and tumor mutation burden (TMB) in different groups: (A) Correlation between the abundance of immune cells and 7 genes in the prognostic signature. (B) Differential analyses of TME score among StromalScore, ImmuneScore, and ESTIMATEScore. (C) The somatic gene mutations in high-risk group. (D) The somatic gene mutations in low-risk group. (E) Differential analysis of TMB in high- and low-risk score groups. (F) Positively correlation analysis of PRG Risk score and TMB (*R* = 0.088, *P* = .0061). (G) Positively correlation analysis between PRG Risk score and CSC index (*R* = 0.17, *P* = 3.8e-08e). *P < .05, **P < .01, ***P < .001. ns = not statistically different, PRGs = PANoptosis-related genes.

Through CIBERSORT algorithm, we conducted correlation analysis to identified the association between PRG risk score and the abundance of 22 immune cells. Supplemental Figure S2A-L, http://links.lww.com/MD/N484 showed that Macrophages M0, Macrophages M2, Mast cells resting, Plasma cells displayed a positive association with the risk score. In contrast, helper B cells memory, B cells naive, Macrophages M1, T cells CD4 memory activated, T cells CD8, T cells follicular helper, and T cells regulatory (Tregs) were negatively correlated with PRG risk score. Furthermore, we also assessed the association between the abundance of immune cells and the 5 hub genes. The correlation results demonstrated significant association between most immune cells and these 5 signature genes (Fig. 8A). The TME scores of stromal, immune, and ESTIMATE score were computed for both the high-risk and low-risk groups. It was observed that the low-risk group had a higher stromal score, immune score, and ESTIMATE score (Fig. 8B).

### 3.7. Association somatic mutations, TMB, and CSC in high- and low-risk group

To investigate the differences in gene mutations between the high- and low-risk group of BC patients, we employed the “maftools” of R package. The analysis revealed that the top 3 mutated genes was TTN, TP53, and MUC16 in both groups (Figs. 8C-D). In addition, the association between TMB and risk score was assessed, and the result showed that risk score was significantly positive associated with TMB, indicating higher risk score group had higher TMB (Figs. 8E-F). Our result revealed a positive linear correlation between PRG Risk score and CSC index values (*R* = 0.17, *P* < .05) (Fig. 8G).

### 3.8. Drugs susceptibility analysis

We conducted a comparative analysis of the low-risk and high-risk groups to assess the predictive efficacy of this signature on therapeutic effects of various drugs for BC. Therapeutic effects in the high- and low- group of various drugs for BC is provided in Supplemental Table S4, http://links.lww.com/MD/N484. Our findings revealed that the low-risk group exhibited significantly higher IC50 values with chemotherapy drugs, including Acetalax, BI-2536, Sepantronium and UMI-77 (Supplemental Figure S3A-D, http://links.lww.com/MD/N484). Conversely, high-risk group demonstrated increased sensitivity to 5-fluorouracil, Afatinib, Carmustine, Cisplatin, Crizotinib, Cyclophosphamide, Cytarabine, Epirubicin, Erlotinib, Fludarabine, Ruxolitinib, Selumetinib, Sinularin, Sorafenib, Staurosporine, Tamoxifen, Telomerase, Temozolomide, Teniposide, Trametinib, and Zoledronate (Supplemental Figure S3E-Y, http://links.lww.com/MD/N484). These results indicate that the PRG Risk score signature has the potential to predict the response of BC patients to specific chemotherapy drugs, thus offering valuable insights for personalized treatment strategies.

### 3.9. qRT-PCR validated the signature genes expression

**Figure 9.**
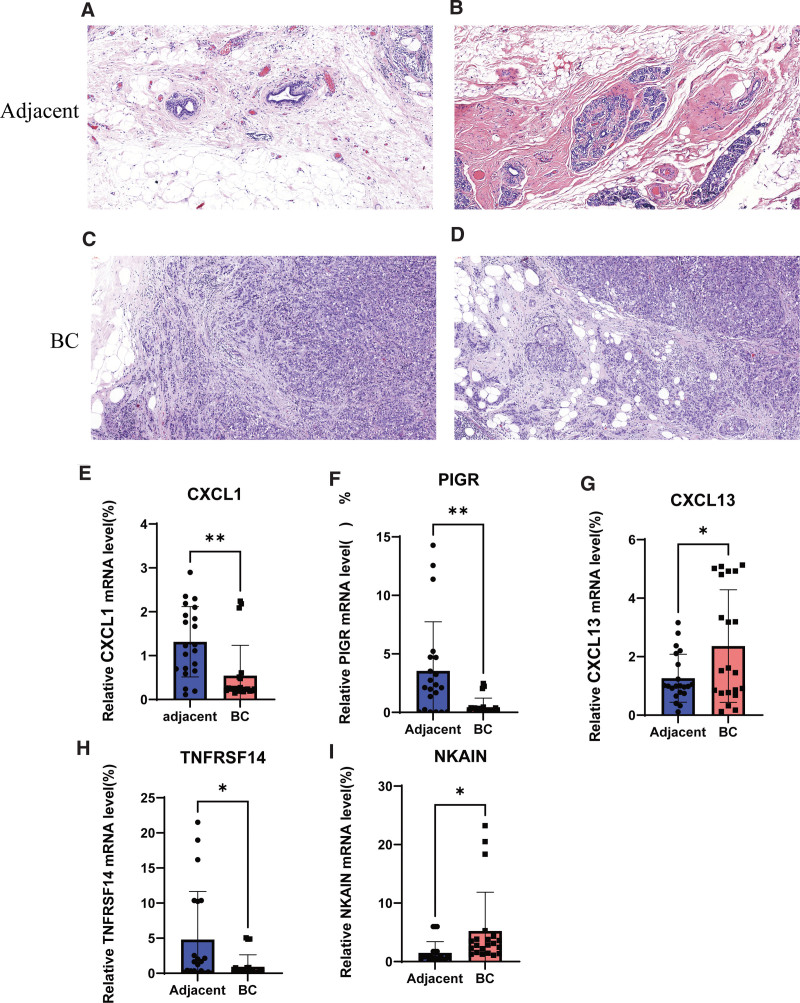
Verify the mRNA expression levels of the 5 signature genes in the tissues: (A–D) HE staining images in BC tissue and adjacent tissues. (E–I) Differential expression of CXCL1, PIGR, CXCL13, TNFRSF14, and NKAIN in adjacent non-tumor tissues and BC tissues. *P < .05, **P < .01, ***P < .001. BC = breast cancer, ns = not statistically different.

Figure 9A-D showed the HE staining of adjacent tumor tissues and BC. BC and adjacent tumor clinical samples were then used to assessed the expression levels of the PRG related prognostic signature genes. The qRT-PCR results showed that CXCL1, PIGR, and TNFRSF14 were significantly lower expressed in BC tissues than adjacent tissue, while CXCL13 and NKAIN were significantly upregulated in BC tissues compared with adjacent tumor tissue (Figs. 9E-I).

## 4. Discussion

The prevalent of BC has surpassed lung cancer to become the most prevalent cancer type among women according to the GLOBOCAN 2020. It revealed that about 2.3 million new cases of BC were occurred in 2020, constituting 11.7% of all cancer cases.^[24]^ While another study reported that BC has estimated to become the most common cancer in United states in 2022, with an estimated number of 429,105 new cases and the burden of BC is steadily increased.^[25]^ Notably, incidence and mortality rates of BC exhibit variations among different ethnicities and races in the United States. American black women exhibit the lowest 5-year relative survival rates across all racial and ethnic groups for each molecular subtype and stage of the disease.^[26]^ The high level of heterogeneity in BC, stemming from variations in genetic status and molecular subtypes, leads to notable differences in patient prognosis. Consequently, it is imperative to identify novel prognostic biomarkers or develop models that can assist in clinical diagnosis and treatment, considering these variations. PANoptosis, is a newly discovered inflammatory programmed cell death, including apoptosis, pyroptosis, and necroptosis. Recent studies have underscored the significant role of PANoptosis in both tumorigenesis and anti-tumor therapies.^[15,27,28]^ Although the role of PANoptosis in various cancers has reported,^[18,29]^ its role in BC have not been well studied and need further exploration.

In this study, we investigated a set of 29 PRGs to explore their somatic mutations, CNVs, disparities in PRG expression between BC and control samples. CNVs represent a common form of somatic mutations within DNA. Our findings unveiled the potential disruptive impact of CNVs on the expression of a majority of PANoptosis genes. Most of PRGs were upregulated in BC cancer, while CASP1 and TAB2 were downregulated in BC patients. Previous study also confirmed that the expression of CASP1 were significant decreased compared with tumor-adjacent tissues(*P* < .05), and CASP1 may contribute to the enhanced proliferation and invasion of BC cells.^[30]^ Activated by TαPcZn-PDT in MCF-7 cells, CASP1 could promoted apoptosis and pyroptosis.^[31]^ Through survival analysis, we found that high expression of CASP1 was associated with better OS rates. Xianghou Xia et al found that the expression of GSDMD in BC was 1.67 times higher compared with normal tissues and was positively associated with OS and relapse free survival, indicating GSDMD was a promising prognostic marker and could be a predictor of therapeutic response for BC.^[32]^ In our study, we also confirmed the finding that GSDMD was upregulated in BC patients and higher expression of GSDMD was correlated with better OS rates.

We initiated a comprehensive analysis by PRGs, resulting in the identification of DEPRGs. Subsequently, through a series of analytical methods including univariate regression, LASSO Cox regression, and multivariate regression, we successfully identified 5 signature genes (CXCL13, TNFRSF14, PIGR, CXCL1, NKAIN1). These 5 signature genes were then used to construct a novel prognostic risk signature, facilitating the identification of potential molecular subtypes for a more accurate prognosis prediction in BC. We found that PRGcluster A and genecluster A were positively associated higher expression of most PRGs, OS rates, and have a lower risk score. These results suggesting that higher expression of most PRGs in BC may achieved a better OS. In addition, we also found that low-risk group was positively associated with higher expression of most PRGs and better OS. The AUC of these signature were all above 0.5. This results together confirmed that our signature could effectively predicted the prognosis of BC patients.

These 5 risk genes have been studied in BC previously. Yuanyuan Zhang et al show that 2 T cell clusters (CD8-CXCL13, CD4-CXCL13) expressed in TNBC tumors responsive to PD-L1 blockade therapy. Of note, T cell exhaustion-related genes of TIGIT, CTLA4, and LAG3 were expressed to CD8-CXCL13. What’s more, CXCL13 + associated B cells also predicted expresses to anti-PD-L1 immunotherapy.^[33]^ Our study found that CXCL3 also correlated with T cells and associated B cells, including Tregs, gamma delta T cells, follicular helper T cells, CD8 T cells, CD4 memory activated T cells and memory B cells. Furthermore, Mast cells resting, Macrophages M2, Macrophages M1 and Macrophages M0 were also correlated with CXCL13. Previous studies have demonstrated the overexpression of CXCL13 in BC patients, implicating its role in BC progression. This suggests that CXCL13 could serve as a valuable diagnostic marker and a potential therapeutic target in BC.^[34–36]^ In our study, CXCL13 was also identified with one of the risk genes which can be predict the diagnosis of BC and we also found the mRNA expression of CXCL13 in BC was higher than that in normal breast tissues. TNFRSF14, also known as TNF receptor superfamily member 14 or HVEM (herpesvirus entry mediator), is a cell surface protein that belongs to the tumor necrosis factor receptor superfamily. This receptor is found on the surface of various immune cells, including T cells, B cells, and dendritic cells.^[37–39]^ Previous study showed that TNFRSF14 is correlated with a favorable prognosis in various cancer patients, its specific role in BC remains unclear.^[38]^ TNFRSF14 serves as a pivotal role in multiple signaling pathways, including NF-κB, AP-1, AKT, RELA, and others, playing a crucial role in immune system function. This includes promoting T-cell co-stimulation, activating autoimmune-mediated inflammatory responses, regulating dendritic cell homeostasis, and enhancing host defense against pathogens.^[37,38]^ The mRNA TNFRSF14 expression was downregulated in BC compared with adjacent tumor tissues, which was consistent with previous study.^[38]^ In addition, we also found that TNFRSF14 was associated with T cells, B cells and NK cells. Polymeric immunoglobulin receptor (PIGR), a key inflammatory mediator, has been identified as a biomarker in various cancers/tumors.^[40]^ Previous study reported that PIGR was downregulated in BC patients and lower expression of PIGR was significant correlated with decreased 5-year survival rate.^[41]^ Wichitra Asanprakit et al demonstrated that Macrophages M1 and M2 can upregulate the expression level of PIGR.^[41]^ Yulong Bao et al found found a significant 32-fold downregulation of PIGR in breast tumor tissues, suggesting that PIGR may have a restraining effect on breast tumor tissues and could potentially be a therapeutic target for treating BC patients.^[42]^ In our research, we confirmed PIGR was associated with Macrophages M2, Macrophages M1 and Macrophages M0. CXCL1, belonging to the CXC chemokine family, has been documented as a pivotal factor in the development of inflammatory diseases and various tumor progression. In BC, the expression of CXCL1 was lower in BC compared with normal tissues, we study also confirmed this result by qRT-PCR. Higher expression of CXCL1 can promote the proliferation of BC and was positively associated with TNM stage, lymph node metastasis.^[43,44]^ Knockdown the CXCL1 in THP-1 cells can not only halted the growth-promoting effects of macrophages but also led to a significant reduction in BC growth. CXCL1 can promote the metastasis of BC through NF-κB/SOX4 signaling. In our study, we confirmed that CXCL1 was significantly associated with Macrophages M1 and M2. NKAIN1 is a mammalian protein belonging to a family of proteins that shares similarities with Drosophila Nkain and interacts with the beta subunit of Na,K-ATPase(Na+/K + pump) and has been implicated in ion transport and regulation of cell volume.^[45]^ Yusha Liu et al identified NKAIN1 as one of the signature genes in BC patients and showed a positive correlated with Macrophage.^[46]^ In our study, we found NKAIN1 was positively associated with Macrophages M2, while negatively associated with Macrophages M1.

We also investigated the correlation between the risk score and immune cells, revealing that 4 types of immune cells displayed a positive association with the risk score, while the other 8 types of immune cells exhibited a negative correlation. Compared with high-risk group, we observed that the low-risk group exhibited higher stromal scores, immune scores, and ESTIMATE scores. This observation suggests that our risk model has the potential to predict the composition of the TME and that individuals in the low-risk groups may respond more positively to anti-tumor therapy.

CSCs have gained recognition as promising therapeutic targets in cancer therapy due to their differentiation potential and self-renewal capacity.^[47]^ Our result found a positive correlation between CSC index and risk score, indicating that BC cells with a higher-risk score exhibit a reduced degree of cellular differentiation and a greater stem cell property.

However, our study has some limitations should be warranted. The data in this study were mainly derived from the TCGA and GEO databases, and most of the data may be retrospective, which may have caused inherent case selection bias. Furthermore, it’s important to note that our validation efforts only encompassed partial expression verification at the tissue level and the BC tissue were not classified into subtypes. To strengthen our findings, additional in vitro and in vivo experiments are essential. Thirdly, this study did not go into the subtypes of breast cancer, and the correlation between the subtypes of breast cancer and PANoptosis still needs further study. Furthermore, the mechanisms responsible for the differential response to immunotherapy between high-risk and low-risk populations remain incompletely understood and warrant further investigation. Finally, it’s worth noting that certain valuable clinical features, including surgery, tumor markers, and neoadjuvant chemotherapy, were not included into our study. Therefore, clinical cases are necessary to validate and confirm our results.

## 5. Conclusion

In summary, we have developed a molecular clustering and prognostic signature based on PANoptosis to improve the prediction of BC prognosis. This signature plays a significant role in predicting survival, understanding the tumor immune microenvironment, and guiding treatment decisions for BC patients. This discovery has the potential to not only assist in assessing overall patient prognosis but also to deepen our understanding of the underlying mechanisms of PANoptosis in BC pathogenesis, offering novel insights for the development of effective treatments.

## Acknowledgments

Thanks to TCGA and GEO for providing a large amount of data.

## Scope statement

Our current study focuses on the role, significance, therapeutic possibilities, and prognostic value of PANoptosis-related genes (PRGs) and pathways in shaping the tumor microenvironment, influencing immune responses, and influencing patient outcomes in BC. The relationship between PANoptosis-related genes (PRGs) and BC studied in this study is highly consistent with the gene research in Frontier in genetics.

## Author contributions

**Data curation:** LinJing Guan, Li Yang.

**Formal analysis:** Yiming Cao, LinJing Guan, Chang-yuan Wei.

**Investigation:** Yiming Cao, Li Yang.

**Methodology:** Yiming Cao.

**Supervision:** Chang-yuan Wei.

**Writing – original draft:** LinJing Guan.

**Writing – review & editing:** Chang-yuan Wei.

## Supplementary Material


