# Supplementary material for: PANoptosis-related molecular clustering and prognostic signature associated with the immune landscape and therapy response in breast cancer
Source: Medicine (Baltimore). 2024 Sep 13;103(37):e39511. doi: 10.1097/MD.0000000000039511 (PMC11404910; doi:10.1097/MD.0000000000039511)

Supplementary Table S1. 29 PRGs according to previous researches.	
**Gene**	**Type**
**CASP8**	PANoptosis
**FADD**	PANoptosis
**CASP6**	PANoptosis
**NLRP3**	PANoptosis
**TAB2**	PANoptosis
**TAB3**	PANoptosis
**PSTPIP2**	PANoptosis
**TNFAIP3**	PANoptosis
**CASP7**	PANoptosis
**PARP1**	PANoptosis
**GSDMD**	PANoptosis
**MLKL**	PANoptosis
**IRF1**	PANoptosis
**AIM2**	PANoptosis
**ZBP1**	PANoptosis
**CASP1**	PANoptosis
**RIPK1**	PANoptosis
**RIPK3**	PANoptosis
**TRADD**	PANoptosis
**ASC**	PANoptosis
**TAK1**	PANoptosis
**MEFV**	PANoptosis
**PYCARD**	PANoptosis
**NAIP2**	PANoptosis
**NAIP6**	PANoptosis
**NLRC4**	PANoptosis
**NAIP5**	PANoptosis
**NLRP1A**	PANoptosis
**CCFLAR**	PANoptosis

Supplementary Table S2. The clinical characteristics of the TCGA cohort and GSE37751 cohort.

Variable	TCGA cohort (N=1097)	GSE37751 cohort (N=61)
Age		
≤65 years	776	48
>65 years	321	13
Sex		
Female	1085	61
Male	12	0
Grade		
G1	0	8
G2	0	20
G3	0	25
G4	0	
unknow	1097	8
Stage		
I	183	4
II	620	43
III	248	14
IV	20	
X	13	
unknow	11	
T classification		
T1	281	NA
T2	635	NA
T3	138	NA
T4	40	NA
TX	3	NA
unknow	0	NA
M classification		
M0	912	NA
M1	22	NA
MX	163	NA
N classification		
N0		33
N1		23
N2		5
unknow		
Survival status(OS)		
Death	948	26
Survival	149	35

Supplementary Table S3. Primer sequences for PCR amplification.		
**Genes**	**Forward Primer**	**Reverse Primer**
CXCL13	5‘-TATCCCTAGACGCTTCATTGATCG-3’	5‘-CCATTCAGCTTGAGGGTCCACA-3’
TNFRSF14	5‘-TTCTCTCAGGGAGCCTCGTCAT-3’	5‘-CTCACCTTCTGCCTCCTGTCTT-3’
PIGR	5‘-TACTGGTGTGGAGTGAAGCAGG-3’	5‘-AGCACCTTCTCATCAGGAGCAG-3’
CXCL1	5‘-AGCTTGCCTCAATCCTGCATCC-3’	5‘-TCCTTCAGGAACAGCCACCAGT-3’
NKAIN1	5‘-TCATCTCTGTCACTGGCTGCCT-3’	5‘-CTCCAGGAACACTTTGCTCACG-3’

Supplementary Table S4. Drug sensitivity analysis		
**Drugs**	**Low risk group(P value)**	**High risk group(P value)**
**5-Fluorouracil**		P<2.22e-16
**ABT737**		5.4e-07
**Acetalax**	0.0001	
**Afatinib**		0.00011
**Afuresertib**		0.00053
**AGI-5198**		P<2.22e-16
**AGI-6780**		1.5e-11
**Alisertib**		P<2.22e-16
**Alpelisib**		3.1e-12
**AMG-319**		P<2.22e-16
**AZ960**		P<2.22e-16
**AZ6102**		P<2.22e-16
**AZD1208**		1.1e-15
**AZD1332**		P<2.22e-16
**AZD2014**		6.5e-08
**AZD3759**		P<2.22e-16
**AZD4547**		7.5e-06
**AZD5153**		P<2.22e-16
**AZD5363**		1.7e-10
**AZD5438**		P<2.22e-16
**AZD5582**		2.1e-05
**AZD5991**		0.00024
**AZD6482**		1.8e-09
**AZD6738**		P<2.22e-16
**AZD7762**		P<2.22e-16
**AZD8055**		P<2.22e-16
**AZD8186**		P<2.22e-16
**BDP-00009066**		P<2.22e-16
**BI-2536**		P<2.22e-16
**BIBR-1532**		1.8e-13
**BMS-345541**		P<2.22e-16
**BMS-536924**		P<2.22e-16
**BMS-754807**		P<2.22e-16
**Bortezomib**		3.1e-15
**BPD-00008900**		P<2.22e-16
**Buparlisib**		P<2.22e-16
**Camptothecin**		P<2.22e-16
**Carmustine**		3.3e-10
**CDK9-5038**		P<2.22e-16
**CDK9-5576**		P<2.22e-16
**Cediranib**		P<2.22e-16

**Cisplatin**		P<2.22e-16
**Crizotinib**		P<2.22e-16
**Cyclophosphamide**		P<2.22e-16
**Cytarabine**		P<2.22e-16
**CZC24832**		P<2.22e-16
**Dabrafenib**		P<2.22e-16
**Dactinomycin**		P<2.22e-16
**Dactolisib**		1.4e-12
**Dasatinib**		P<2.22e-16
**Dlhydrorotenone**		0.0005
**Dinaciclib**		P<2.22e-16
**Docetaxel**		P<2.22e-16
**Eg5-9814**		1.1e-14
**Elephantin**		2e-08
**Entinostat**		3.5e-11
**Entospletinib**		P<2.22e-16
**Epirubicin**		P<2.22e-16
**EPZ004777**		P<2.22e-16
**EPZ5676**		P<2.22e-16
**ERK-2440**		5e-15
**ERK-6604**		P<2.22e-16
**Erlotinib**		3.5e-12
**Fludarabine**		P<2.22e-16
**Foretinib**		P<2.22e-16
**Fulvestrant**		1.5e-10
**Gallibiscoquinazole**		2.1e-07
**GDC0810**		4.5e-08
**Gefitinib**		1e-11
**Gemcitabine**		P<2.22e-16
**GNE-317**		P<2.22e-16
**GSK343**		P<2.22e-16
**GSK591**		P<2.22e-16
**GSK269962A**		P<2.22e-16
**GSK1904529A**		1.1e-06
**GSK2578215A**		3.9e-10
**GSK2606414**		P<2.22e-16
**IAP-5620**		3e-08
**I-BET-762**		P<2.22e-16
**I-BRD9**		8.4e-13
**Ibrutinib**		2.8e-10
**IGF1R-3801**		P<2.22e-16
**IRAK4-4710**		P<2.22e-16
**Irinotecan**		P<2.22e-16

**IWP-2**		P<2.22e-16
**JAK-8517**		P<2.22e-16
**JAK1-8709**		P<2.22e-16
**JQ1**		P<2.22e-16
**KRAS(G12C)**		P<2.22e-16
**KU-55933**		P<2.22e-16
**Leflunomide**		P<2.22e-16
**LGK974**		P<2.22e-16
**Linsitinib**		1e-09
**LJI308**		P<2.22e-16
**Luminespib**		P<2.22e-16
**LY2109761**		P<2.22e-16
**MG-132**		1.3e-10
**MIM1**		6.7e-16
**MIRA-1**		4.1e-15
**Mirin**		P<2.22e-16
**Mitoxantrone**		P<2.22e-16
**MK-1775**		P<2.22e-16
**MK-2206**		2.4e-09
**MK-8776**		1.5e-15
**ML323**		0.00011
**MN-64**		2.9e-12
**Navitoclax**		3.2e-06
**Nelarabine**		P<2.22e-16
**Niraparib**		P<2.22e-16
**NU7441**		1.9e-06
**Nutlin-3a(-)**		5e-15
**NVP-ADW742**		3.4e-11
**Obatoclax Mesylate**		P<2.22e-16
**OF-1**		1.6e-06
**Olaparib**		P<2.22e-16
**Osimertinib**		2.3e-06
**OTX015**		P<2.22e-16
**Oxaliplatin**		P<2.22e-16
**Paclitaxel**		1.3e-10
**Pak-5339**		P<2.22e-16
**Palbociclib**		P<2.22e-16
**PCI-34051**		P<2.22e-16
**PD173074**		P<2.22e-16
**PD0325901**		P<2.22e-16
**Pevonedistat**		P<2.22e-16
**PF-4708671**		P<2.22e-16
**PFI3**		P<2.22e-16

**Picolinici-acid**		P<2.22e-16
**Pictilisib**		2.5e-14
**PLX-4720**		P<2.22e-16
**Podophyllotoxin bromide**		P<2.22e-16
**PRIMA-1MET**		1.3e-13
**PRT062607**		P<2.22e-16
**Pyridostatin**		4.1e-10
**Rapamycin**		P<2.22e-16
**Ribociclib**		P<2.22e-16
**RO-3306**		9.1E-05
**Ruxolitinib**		P<2.22e-16
**RVX-208**		P<2.22e-16
**Sabutoclax**		9.4e-14
**Sapitinib**		19e-07
**Savolitinib**		1.2e-10
**SB216763**		P<2.22e-16
**SB505124**	P<2.22e-16	
**SCH772984**		P<2.22e-16
**Selumetinib**		P<2.22e-16
**Sepantronium**	4.4e-11	
**Sinularin**		7.3e-07
**Sorafenib**		1.4e-09
**Staurosporine**		P<2.22e-16
**TAF1-5496**		0.0001
**Talazoparib**		P<2.22e-16
**Tamoxifen**		2.7e-07
**Taselisib**		P<2.22e-16
**Telomerase**		6e-09
**Temozolomide**		P<2.22e-16
**Teniposide**		P<2.22e-16
**Topotecan**		P<2.22e-16
**Tozasertib**		2.9e-12
**Trametinib**		P<2.22e-16
**Ulixertinib**		1.7e-15
**UMI-77**	0.0002	
**Uprosertib**		9.5e-05
**VE821**		P<2.22e-16
**VE-822**		P<2.22e-16
**Venetoclax**		P<2.22e-16
**Vinblastine**		P<2.22e-16
**Vincristine**		P<2.22e-16
**Vinorelbine**		P<2.22e-16
**Vorinostat**		3.2e-05

**VSP34-8731**		P<2.22e-16
**VX-11e**		P<2.22e-16
**Wee1 Inhibitor**		8.9e-09
**WEHI-539**		6.3e-09
**WIKI4**		P<2.22e-16
**Wnt-C59**		P<2.22e-16
**WZ4003**		P<2.22e-16
**XAV939**		P<2.22e-16
**YK-4-279**		P<2.22e-16
**ZM447439**		P<2.22e-16
**Zoledronate**		P<2.22e-16

Supplemental Figure S1: β-actin gene analysis of Carcinoma cDNA template by agarose gel electrophoresis.Lane M :DNA ladder; Lanes 1-21 : 21 Carcinoma samples cDNA.

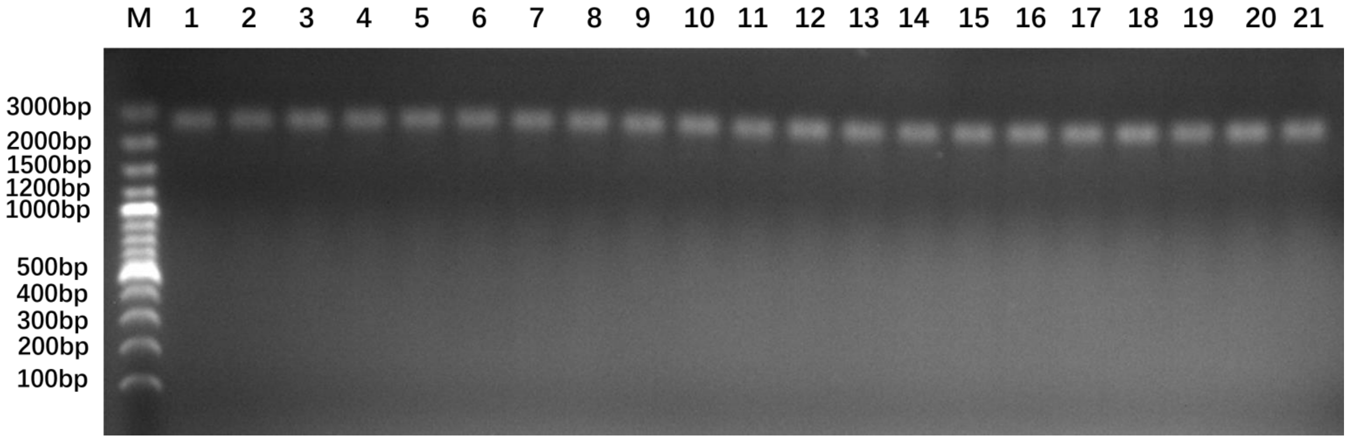

Supplemental Figure S2: Assessment of the tumor microenvironment between two risk subgroups. (A) Relevance between PRG risk score and B cells memory. (B) Relevance between PRG risk score and B cells naive. (C) Relevance between PRG risk score and Dendritic cells resting. (D) Relevance between PRG risk score and Macrophages M0. (E) Relevance between PRG risk score and Macrophages M1. (F) Relevance between PRG risk score and Macrophages M2. (G) Relevance between PRG risk score and Mast cells resting. (H) Relevance between PRG risk score and Plasma cells. (I) Relevance between PRG risk score and T cells CD4 memory activated. (J) Relevance between PRG risk score and T cells CD8. (K) Relevance between PRG risk score and T cell follicular helper. (L) Relevance between PRG risk score and T cells regulatory (Tregs).

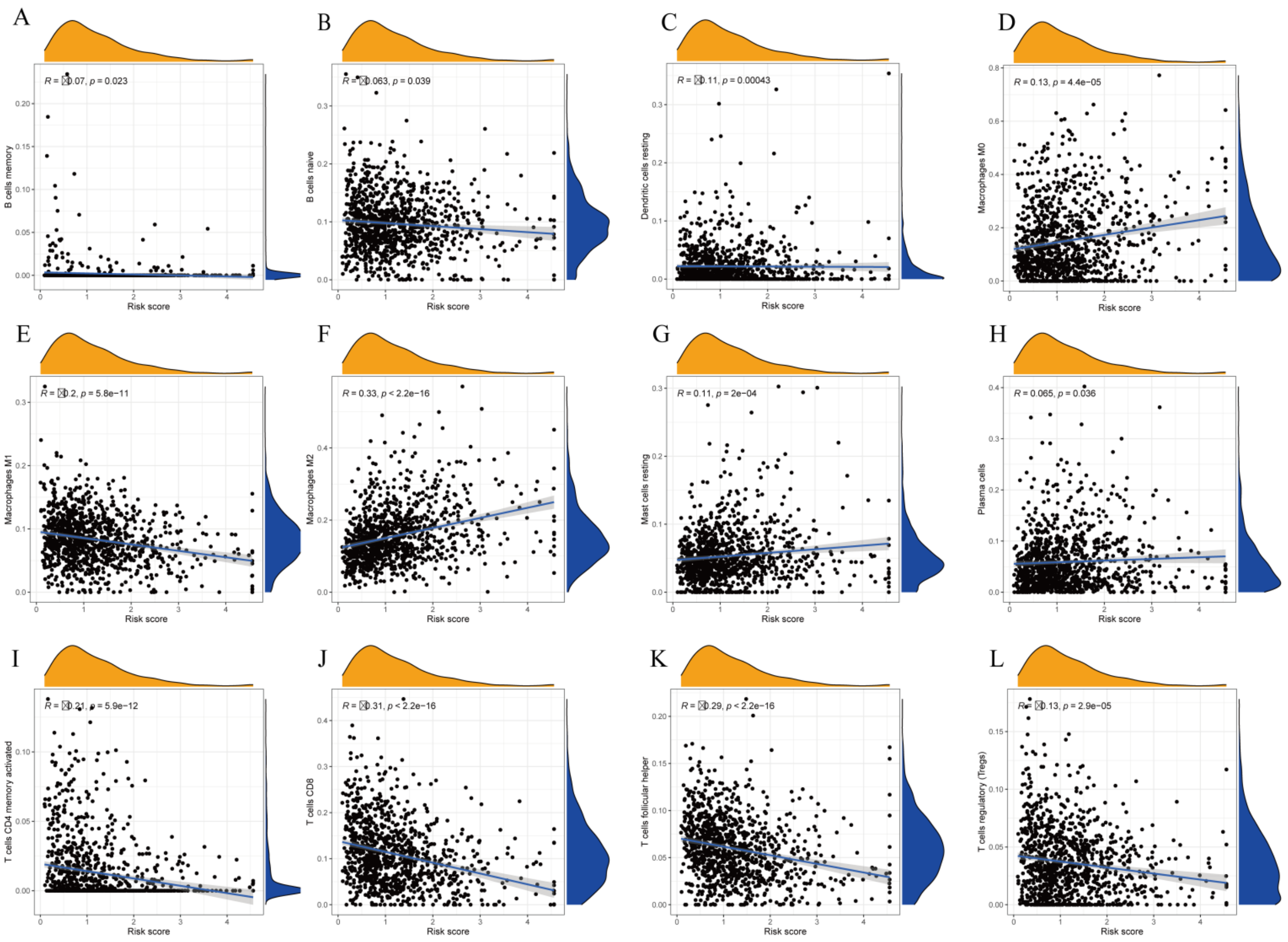

Supplemental Figure S3: Exploration of potential chemotherapeutic compounds for breast cancer in the high- and low-risk subgroups: The drug sensitivity evaluation of (A) Acetalax, (B)BI-2536,(C) Sepantronium, (D)UMI-77, (E) 5-fluorouracil, (F)Afatinib, (G)Carmustine, (H)Cisplatin, (I)Crizotinib, (J)Cyclophosphamide, (K)Cytarabine, (L)Epirubicin, (M)Erlotinib, (N)Fludarabine, (O)Ruxolitinib, (P)Selumetinib, (Q)Sinularin, (R)Sorafenib, (S)Staurosporine, (T)Tamoxifen, (U)Telomerase, (V)Temozolomide, (W)Teniposide, (X)Trametinib, and (Y)Zoledronate.

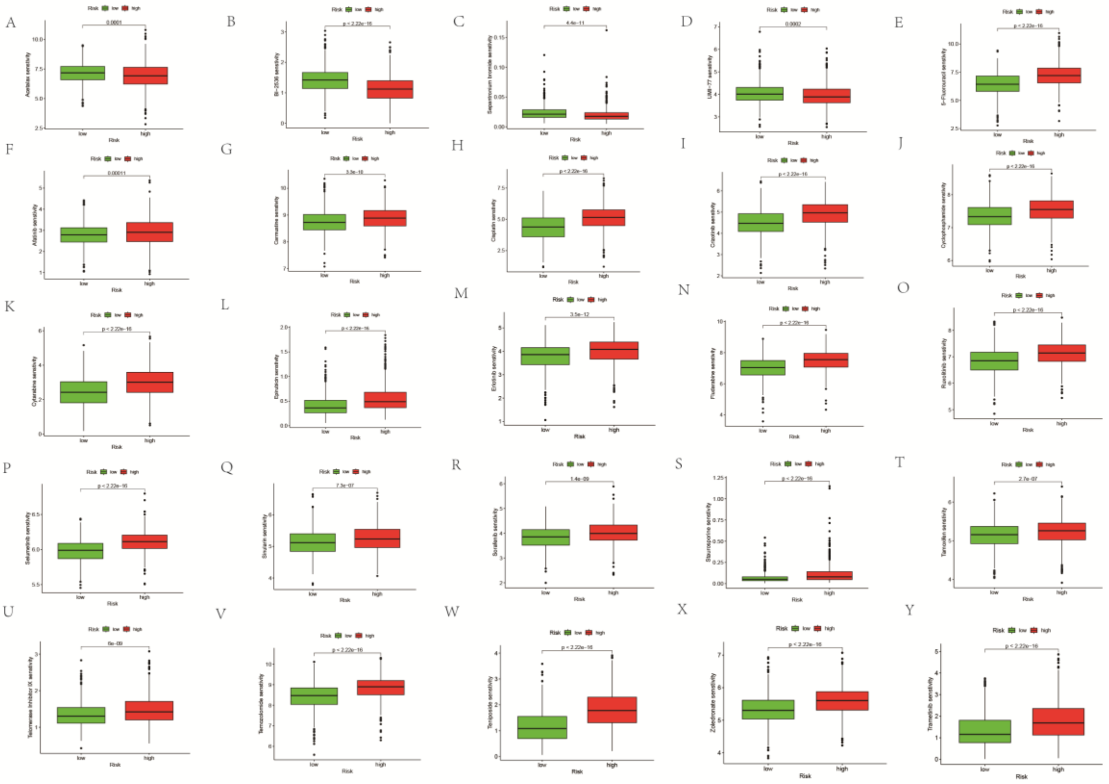

Supplement: Supplementary file 1 [file medi-103-e39511-s001.pdf]
